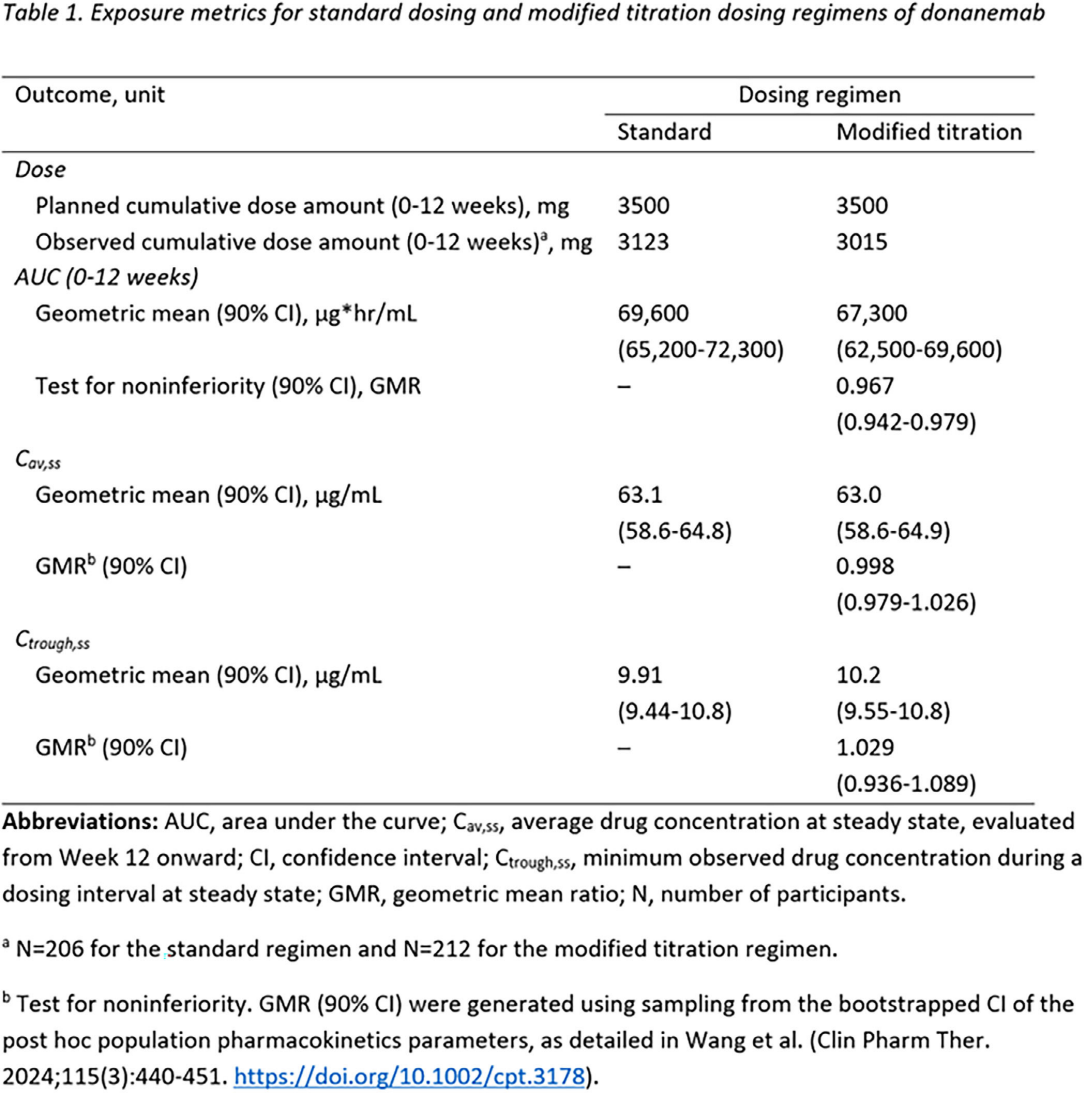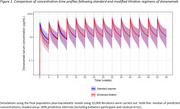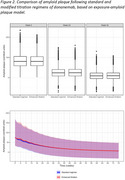# Comparable cumulative exposure and amyloid reduction following donanemab standard and modified titration dosing in participants with early symptomatic Alzheimer’s disease from TRAILBLAZER‐ALZ 6

**DOI:** 10.1002/alz70861_108108

**Published:** 2025-12-23

**Authors:** Ivelina Gueorguieva, Kay Hoong Chow, Sergey Shcherbinin, Hong Wang, Emel Serap Monkul Nery, Dawn A. Brooks, John R. Sims

**Affiliations:** ^1^ Eli Lilly and Company, Bracknell, Berkshire UK; ^2^ Eli Lilly and Company, Indianapolis, IN USA

## Abstract

**Background:**

Donanemab is an antibody approved for the treatment of Alzheimer's disease. TRAILBLAZER‐ALZ 6 (NCT05738486) assesses the impact of different donanemab dosing regimens on the frequency of amyloid‐related imaging abnormalities‐edema/effusions in relation to amyloid reduction in participants with early symptomatic Alzheimer’s disease. A previously reported pharmacokinetic/pharmacodynamic (PK/PD) model was used to design TRAILBLAZER‐ALZ 6 ensuring similarity in cumulative exposure and amyloid plaque reduction across study arms. The effectiveness of a new dosage regimen for an approved drug may be inferred by demonstrating bridging, similarity/non‐inferiority in systemic exposures. Here we evaluate similarity in donanemab exposure and amyloid‐lowering effects between TRAILBLAZER‐ALZ 6 standard dosing and modified titration regimens over 52 weeks.

**Methods:**

In this multicenter, double‐blind, phase 3b study, participants on the standard regimen (700 mg monthly for first three doses, followed by 1400 mg monthly) and modified regimen (350 mg, 700 mg, and 1050 mg monthly for first three doses, followed by 1400 mg monthly) received donanemab until meeting amyloid plaque clearance criteria or Week 76. Serum PK exposure metrics were measured, and positron emission tomography was used to assess changes in brain amyloid plaque deposition from baseline to Week 52. Population PK/PD analyses in NONMEM® and non‐inferiority assessment for exposure were utilized to analyze data up to Week 52.

**Results:**

The planned and observed cumulative doses and cumulative area under the curve (AUC 0‐12 weeks) were similar for the standard (*N* =206) and modified (*N* =212) regimens (Table 1). The modified regimen was noninferior in exposure to the standard regimen. Over 52 weeks, both regimens achieved similar cumulative doses and exposures and provided consistent C_av,ss_ associated with amyloid plaque reduction (Figure 1). At Weeks 24 and 52, the distributions of amyloid plaque reduction of both regimens largely overlapped (Figure 2). PK/PD model‐based extrapolation to Week 76 showed the regimens continued to overlap (Figure 2).

**Conclusions:**

The standard and modified dosing arms in TRAILBLAZER‐ALZ 6 achieved similar cumulative doses and exposures (AUC) and provided consistent C_av,ss_ associated with amyloid plaque reduction. Bridging based on PK/PD for these two regimens was ascertained.